# Cobalt exposure triggers impairments in cognitive and anxiety-like behaviors, brain oxidative stress and inflammation, and hippocampo-amygdala histomorphological alterations: Protective role of aqueous *Prosopis africana* seed extract

**DOI:** 10.22038/IJBMS.2022.65689.14456

**Published:** 2022-12

**Authors:** Rademene Sunday Oria, Runyi Bassey Ben, Ugochukwu Godfrey Esomonu, Precious Ibiang Essien, Linda Eze Odinaka, Gift Ekligbor Ettah, Otu Otu Eyong, Omamuyovwi Meashack Ijomone

**Affiliations:** 1 Department of Human Anatomy, Faculty of Basic Medical Sciences, Cross River University of Technology (CRUTECH), Okuku Campus, Cross River, Nigeria; 2 The Neuro- Lab, School of Basic Medical Sciences, Federal University of Technology, Akure, Nigeria; 3 Department of Human Anatomy, School of Basic Medical Sciences, Federal University of Technology, Akure, Nigeria

**Keywords:** Amygdala, Cobalt, Hippocampus, Neuroinflammation, Neurotoxicity, Oxidative stress, * Prosopis africana*

## Abstract

**Objective(s):**

Cobalt toxicity has become a health concern in recent years, due to overexposure resulting in neurological impairments. With a growing interest in the therapeutic roles of herbs, in toxicity research, it’s worth looking into the curative effects of aqueous *Prosopis africana* seed extract, a plant rich in flavonoids on cobalt-induced neurotoxicity.

**Materials and Methods:**

We treated rats with CoCl_2_ or CoCl_2_ in combination with aqueous PA seed extract (PAE) orally for 14 days. Control rats received distilled water for the same period. Following treatments, behavioral experiments, analysis for oxidative stress, inflammation, and histological and immunohistochemical analysis were performed.

**Results:**

Results revealed that CoCl_2_ reduced the exploration time, recognition index in the novel object recognition test, percentage spontaneous alternation in the Y-maze tests, and reduced open arm entry and duration in elevated plus-maze. However, treatment with PAE improved these parameters to levels comparable with those of the control group. Furthermore, PAE therapy reduced CoCl_2_-induced surge in hydrogen peroxide, malondialdehyde, TNF-α and IL-1β levels in brain homogenate, while also increasing superoxide dismutase and reduced reduced-glutathione activities. CoCl_2_ exposure resulted in obvious features of neurodegeneration like nuclear disintegration, nuclear shrinkage, and cytoplasmic vacuolations of the cells of the hippocampus and amygdala, with an increased expression of GFAP. The hippocampal and amygdala histology improved after PAE administration, while exacerbated GFAP expressions were attenuated.

**Conclusion:**

These findings imply that PAE may be anxiolytic and can help reduce cognitive impairments and hippocampal damage caused by CoCl_2_ neurotoxicity, via mechanisms that involve attenuation of oxidative stress and inflammation.

## Introduction

Neurotoxicity is among the most common causes of neurodegenerative diseases, which can lead to cognitive degradation, impaired memory (both short and long-term), depressed mood, and the development of psychotic illnesses (1, 2). Heavy metals are frequently associated with the etiology of a variety of neurological ailments due to their neurotoxic impact. Cobalt (Co) is most prevalent in living tissues when it is combined with vitamin B12 (cobalamin). More so, cobalt is an essential component of vitamin B12 and is highly valuable in little doses (3).

However, since cobalt is ubiquitous in the earth’s crust, humans and animals may be exposed to high cobalt quantities in contaminated food and water. Boreholes and shallow (hand-dug) wells, as well as the few surface water bodies in the area, are used by the majority of the population for home, farming, and commercial reasons. As a result, the concentrations of Co in local soil and water bodies exceed accepted safe levels (4). Cobalt exposure is fast becoming a prevalent health concern. Overexposure scenarios include artificial limb (prosthesis) users, workers who use hard-cutting tools, miners, and athletes. Curiously, cobalt is capable of inducing toxicity in biological tissues by enhancing the Hypoxia-inducible factor HIF1a, culminating in hypoxia and eventual oxidative stress by the production of reactive oxygen species (ROS) (5). Whenever ROS concentrations rise beyond the brain’s natural enzymatic anti-oxidant mechanisms, like superoxide dismutase (SOD), catalase (CAT), and glutathione peroxidase (GPX), or reduced glutathione (GSH), can degrade, neuronal damage occurs. Several new studies have focused on low-cost, readily available nutritional approaches for reducing the negative effects of heavy metal exposure (6). Oxidative stress plays a significant role in the etiology of neuronal disorders and neurodegenerative illnesses. In cells like neurons, cobalt exposure at high levels has a high risk of triggering cell damage and metabolic abnormalities. Effects of cobalt chloride toxicity on different tissues have been investigated by different authors, like hepatotoxicity (7, 8), nephrotoxicity (9), reproductive toxicity (7), and cardiotoxicity (10).

*Prosopis africana* (*PA*) is found in North, Central, and West Africa. The fermented seeds of *PA* are well-known as a local seasoning (11). Almost every part of the tree has medicinal value. In Mali, for instance, the leaves, bark, and roots of this plant are used to cure bronchitis, dermatitis, tooth rot, diarrhea, malaria, and stomach pains. *PA* seeds are used to manufacture Daddawa and Okpeye, both of which are used as food condiments in Nigeria. Previous phytochemical investigations have discovered bioactive compounds like phlobatannin, flavonoids, polyphenols, tannins, saponins, steroids, and alkaloids in *PA* seed and pod extracts (12, 13). Flavonoids are organic chemicals present in a variety of plants, including *PA* (12). By neutralizing oxygen radicals, lipid peroxidation, and metal ion sequestration, flavonoids have been shown to protect against oxidative injury (14). 

The goal of this study was to provide an insight into the efficacy of aqueous* PA* seed extract, which is rich in flavonoids, in reversing cognitive and anxiety deficits, oxidative stress, and inflammation, as well as microstructural changes in the hippocampus and amygdala caused by cobalt exposure in adult Wistar rats.

## Materials and Methods

***Chemicals and kits***

cobalt chloride hexahydrate (CoCl_2_·6H2O; #7791-13-1) was obtained from Sigma Chemicals (St Louis, MO, USA). Glial fibrillary acidic protein - GFAP (#16825-1-AP) antibody was obtained from Thermo Fisher, USA. Tumor necrosis factor - TNF-α (#E-EL-R2856) and interleukin 1 beta - IL-1β (#E-EL-R0012) ELISA kits were obtained from Elabscience®, United Kingdom. All of the other chemicals were of the highest quality commercially available.

***Plant material and aqueous extract preparation***

The *PA* seeds were harvested from a local farm near the Cross River State University’s (CRUTECH), Faculty of Basic Medical Sciences, Okuku. The seeds were first collected from the pods of the *PA* tree. They weighed 600 g and were boiled for 5 to 7 hr in a gas cooker before being allowed to cool to room temperature. The goal of boiling was to loosen the hulls and make removing and separating the cotyledons easier. The cotyledons were dehulled and boiled for 10 min before draining through a raffia basket. They were allowed to cool and left to ferment for four days, as reported Uzodinma *et al*. (15). After being sun-dried to a constant weight, the fermented seeds were crushed into *Prosopis* seed powder (16)**. **A soxhlet device was used to extract 100 g of this powder for 18 hr after it had been suspended in 500 ml of distilled water. After being filtered through coarse sieve filter paper, the aqueous extract turned out to have a deep brown color. The filtrate was then lyophilized after being dried under decreased pressure. Until it was utilized, it was kept at a temperature of about 0 to 4 °C.

***Approval of ethical principles***

The research and ethics committee of the Faculty of Basic Medical Sciences at CRUTECH Okuku campus approved this study (CRUT/FBMC/REC/21/012). All animals were treated according to the Guidelines prepared by the National Research Council for the Care and Use of Laboratory Animals (17).

***Animals and experimental design***

For this investigation, we procured sixty male Wistar rats (150 – 200 g) from the Experimental Animal Unit, Faculty of Basic Medical Sciences, CRUTECH. They were kept in clean plastic cages at ambient temperature in a clean environment with a natural day/light cycle. The animals were given unlimited access to a regular laboratory rat diet and water. The rats were divided into four groups of 15 rats each and given the following treatments:

**Control group**: Received distilled water only. 

**CoCl**_2_ **alone group: **Received 40 mg/kg cobalt chloride (CoCl_2_) dissolved in distilled water for 14 days. 

**PAE 50 + CoCl**_2_ **group: **Received CoCl_2_ at 40 mg/kg + *PA* seed extract (PAE) at 50 mg/kg for 14 days.

**PAE 100 + CoCl**_2_ **group: **Received CoCl_2_ at 40mg/kg + *PA* seed extract (PAE) at 100 mg/kg for 14 days.

Administration of both CoCl_2_ and PAE was through oral gavage. The dosages of CoCl_2_ and PAE were based on a previous study (18).

***Neurobehavioural tests***

Behavioral assessments were carried out 24 hr after the last dose. The tests were videotaped in real-time with a digital camcorder and assessed by trained observers blinded to the treatment protocols. The following tests were carried out:

***Novel object recognition test (NORT)***

The NORT is used to examine the hippocampal-dependent recognition memory of rodents. The NORT is based on animals’ natural preference for interacting with novel objects rather than familiar ones. Each mouse was allowed to freely roam the open-field environment (a square arena 80 by 80 cm), consisting of three phases: habituation, familiarization, and test (19). Objects were absent throughout the habituation period. During the familiarization stage, each mouse was placed for 5 min in a box containing two identical objects. After 24 hr, the animals’ recognition memory was assessed by exposing them to one familiar and one unfamiliar object. The amount of time spent sniffing and probing each object was recorded. Object exploration implied that the animal contacted the object either with its nose or forepaws from a distance of 2 cm (20). As stated above, it was not acceptable for the animal to revolve around the object without proper exploration. During the trial, olfactory signals and dirt were eliminated on the device and objects by cleaning them with 20% ethanol. The recognition index (%) was calculated as the ratio of the time spent exploring the novel object and the total time spent exploring both novel and familiar objects multiplied by 100 (19, 21). 

***Y-maze***

The y-maze test battery was carried out as previously described by other researchers (22-24). This test is used to assess the rats’ short-term spatial memory. The animals were placed in a Y-maze with arms that were 75 cm long and 15 cm wide, with a 120° angle between them. The rats were placed on a designated start arm and were free to roam around for 5 min in the maze. The score was based on arm entry (both hind limbs are entirely in the arm). Correct alternation was determined when the animal successfully navigated each maze’s three arms in each triad of exploration (i.e., entering all three arms in the overlapping triplet sets) (e.g., XYZ, ZXY, or YZX). It was regarded as an erroneous alternation after exploring two arms per triad of exploration (e.g., XYX, ZXZ, and YXY). The percentage of spontaneous alternation was computed using the following formula: (successive triplet sets/(total number of arm entries – 2) × 100. To eliminate possible bias due to the smell left by the preceding animal, the apparatus used for this experiment was cleaned with 20% ethanol.

***Elevated plus maze (EPM)***

The EPM is often used to assess anxiety-like behavior in laboratory animals (23, 25). Two open arms (50 x 10 cm) are crossed by two closed arms of the exact dimensions with 30 cm high walls. The “arms” were joined with a central square (10 x 10 cm). The maze is 60 cm above ground level. Each rat was placed in the elevated plus maze in the center, facing an open arm, and given 5 min to explore. The following four variables were assessed: open-arm entries, time spent in open arms, closed-arm entries, and time spent in closed arms. When the hind paws of the rats are entirely within the arm, this is known as an arm entry (26). The apparatus is cleaned with 20% ethanol before testing a new animal to eliminate possible bias due to the smell left by the previous animal.

At the end of all neurobehavioural tests, rats were euthanized. Brains were rapidly excised and processed for either biochemical assays or histological analysis. 

***Biochemical assays***

The brains (from five rats) were separated and washed with 1.15 percent KCl. Whole-brain samples were homogenized in 50 mM Tris–HCl buffer (pH 7.4) containing 1.15 percent potassium chloride and centrifuged for 10 min at 4 °C using a cold centrifuge at 12,000 x g. The biochemical parameters were determined using the supernatant obtained. The activity of SOD was determined using Martin *et al*’s technique (27). GSH level was measured in a solution comprising tissue homogenates, and was carried out according to the related protocols (28). More so, lipid peroxidation was measured as malondialdehyde (MDA) (29). The concentration of hydrogen peroxide (H_2_O_2_) was quantified spectrophotometrically at 560 nm (30). Interleukin 1 beta (IL-1β) and tumor necrosis factor-alpha (TNF-α) concentrations in the brain homogenates were measured using commercially available enzyme-linked immunosorbent assay (ELISA) kits (Elabscience®, UK) according to the manufacturer’s instructions.

***Histological studies***

The remaining brain tissues were fixed in a 10% neutral buffered formalin solution. After fixation, tissues were processed via routine paraffin embedding, and serial sections of 5 μm thickness were produced on a rotary microtome. Tissues were stained using routine Haematoxylin and Eosin (H&E) techniques for general histological appearance as previously described by (31).

***Immunohistochemistry***                                                       

From paraffin-embedded brain samples, thin slices of 5 μm thickness were produced. After deparaffinization, slices were incubated in a citrate-based solution with a pH of 6.0 for heat-mediated antigen retrieval. In 0.3 percent hydrogen peroxide, endogenous peroxidase was blocked. The sections were then treated in primary rabbit antibodies: GFAP (ThermoFisher, USA; #16825-1-AP) at 1:10000 overnight at 4 °C. ImmPRESS^TM^ HRP Anti-Rabbit IgG (Peroxidase) Polymer Reagent (Vector® #MP-7401) was used for secondary incubation. Color development was done using a DAB Peroxidase (HRP) Substrate Kit (Vector® #SK-4100), and sections were counterstained with Harris hematoxylin.

***Image analysis and cell count***

Photomicrographs of the sections were taken using a digital brightfield microscope. At x400 magnification, non-overlapping micrographs were produced and used for image analysis in Image J software (NIH, USA). Immunoreactivity was measured by counting positive immunoreactive cells using the Cell Counter function in the Image J program (32, 33).

***Statistical analysis***

GraphPad Prism software (Version 9, GraphPad Inc., USA) was used to conduct the statistical analysis. Data were evaluated with a one-way Analysis of variance (ANOVA), and Tukey’s test was used for multiple comparisons. At *P*<0.05, results were deemed statistically significant.

## Results

***Novel object recognition (NOR) test***

**Figure 1:**
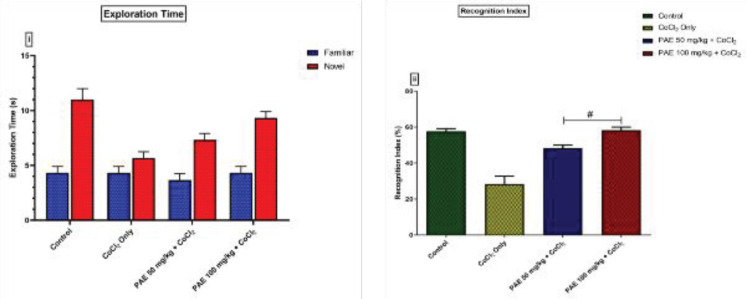
Effect of PAE on short-term memory in novel object recognition (NOR) Test (i) exploration time, (ii) recognition Index. # - significantly different from CoCl_2_ only treated group (*P*<0.05). One-way ANOVA followed by Tukey *post hoc* test. Bars represent means ± S.D; (n=5)

In the NOR test, the cobalt-only treated group showed a decline in the recognition index. However, treatment with either PAE 50 or 100 mg/kg significantly (*P*<0.05) increased the recognition index of these groups when compared with the CoCl_2_-only treated group (Figure 1ii). Furthermore, cobalt-treated rats showed no likeness toward the novel object, although the PAE-treated group explored the novel object more than the familiar object. However, no significant differences in total exploration time of novel vs familiar objects were observed across all groups (Figure 1i).

**Y-Maze**

**Figure 2:**
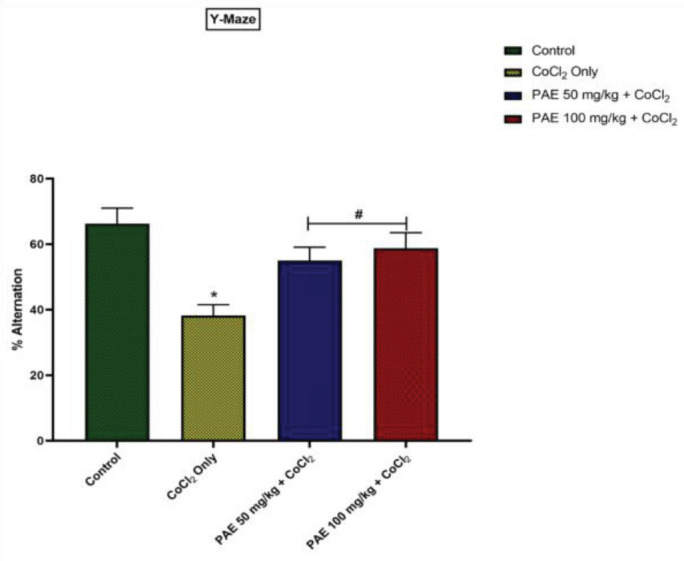
Effect of PAE on % alternation in Y-Maze Test. * - significantly different from Control group (*P*<0.05), # - significantly different from CoCl_2_ only treated group (*P*<0.05). One-way ANOVA followed by Tukey *post hoc *test. Bars represent means ± S.D (n=5)

In the Y-maze test, the percentage of correct alternation was significantly lower in the Cobalt-only treated rats than in the control group (*P*<0.05) (Figure 2). When animals were given a combination of PAE and CoCl_2_, however, there was a significant increase in correct alternation as compared with rats given only CoCl_2_ (*P*<0.05) (Figure 2).

***Elevated plus maze (EPM)***

**Figure 3:**
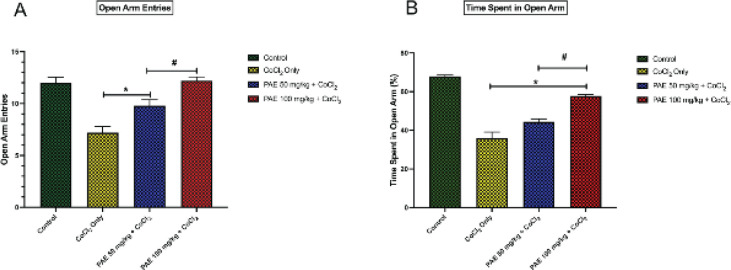
Effect of PAE on anxiety-like behavior in elevated plus maze test. * - significantly different from control group (*P*<0.05), # - significantly different from CoCl_2_ only treated group (*P*<0.05). One-way ANOVA followed by Tukey *post hoc* test. Bars represent means ± S.D; (n=5)

In the elevated plus-maze paradigm, there was a significant difference (*P*<0.05) in all the test parameters namely: open-arm entries and time spent in open arms. Multiple comparisons revealed that there was a significant (*P*<0.05) increase in the open arm entries in PAE 50 and 100 groups when compared with the CoCl_2_-only groups. (Figure 3A). Similarly, there was a significant increase in the %time spent in the open arm of animals in PAE 50 and 100 groups in comparison with the CoCl_2_ only (*P*<0.05) (Figure 3B). 

***Oxidative stress markers***

**Figure 4:**
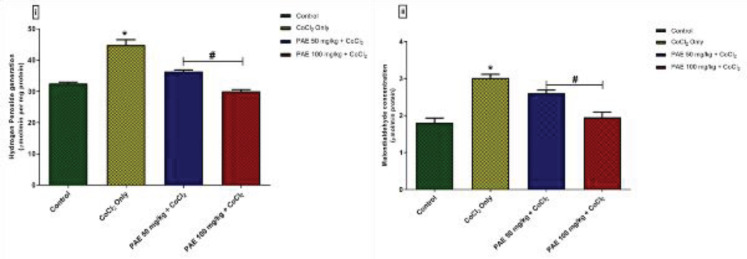
Effect of PAE on oxidative stress markers in the hippocampus of rats exposed to cobalt chloride (i) Hydrogen peroxide (ii) Malondialdehyde. *signiﬁcantly different from the control group (*P*<0.05), #Signiﬁcantly different from Cobalt only group (*P*<0.05). One-way ANOVA followed by Tukey *post hoc* test. Bars represent means ± SD. (n=5)

Assessments of oxidative stress markers such as hydrogen peroxide (H_2_O_2_) and malondialdehyde (MDA) were utilized to evaluate the oxidative alterations caused by CoCl_2_. (Figures 4 i and ii). Compared with the control group, CoCl_2_ administration resulted in a significant increase (*P*<0.05) in brain concentrations of H_2_O_2_ and MDA. Compared with rats exposed to CoCl_2_ alone, concurrent administration of PAE resulted in a considerable decrease in brain levels of H_2_O_2_ and MDA concentrations to levels similar to that of the control.

***Anti-oxidant enzymes status***

**Figure 5:**
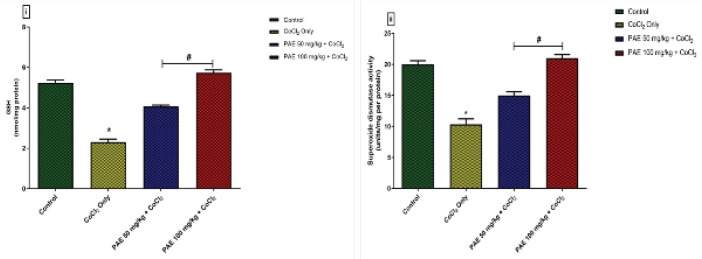
Effect of PAE on antioxidant enzyme activities in the hippocampus of rats exposed to cobalt chloride (i) reduced glutathione, (ii) superoxide dismutase. *signiﬁcantly different from the control group (*P*<0.05), #Signiﬁcantly different from Cobalt only group (*P*<0.05). Bars represent means ± SD. One-way ANOVA followed by Tukey’s *post hoc* test; (n=5)

The activities of anti-oxidant enzymes, like GSH and SOD, were measured for signs of oxidative alterations. When compared with the control group, CoCl_2_ administration resulted in a significant reduction (*P*<0.05) in GSH and SOD activities (Figures 5i and ii). However, PAE therapy boosted the activities of these natural anti-oxidant enzymes in the rats’ brain tissue, and their levels were significantly (*P*<0.05) higher than in CoCl_2 _only group.

***Proinflammatory cytokines***

**Figure 6:**
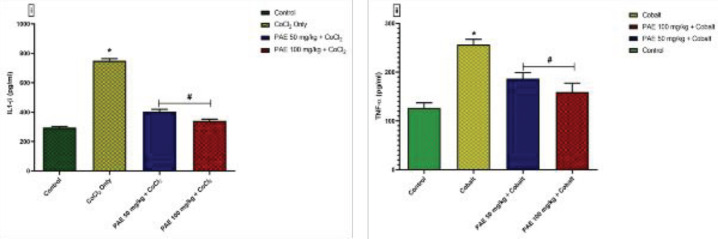
Effect of PAE on serum levels of proinflammatory cytokines of rats exposed to cobalt chloride (i) IL1-β (ii) TNF-α. *signiﬁcantly different from the control group (*P*<0.05), #Signiﬁcantly different from Cobalt only group (*P*<0.05). One-way ANOVA followed by Tukey’s *post hoc *test. Bars represent means ± SD; (n=5)

When compared with the control group, CoCl_2_ administration resulted in a significant rise (*P*<0.05) in brain levels of proinflammatory cytokines, Interleukin 1 beta (IL-1β), and tumor necrosis factor-alpha (TNF-α) (Figures 6i and ii). Co-administration with PAE on the other hand, resulted in a significant (*P*<0.05) reduction in these cytokines when compared with CoCl_2_ only group.

***Histology***

*Hippocampus*

**Figure 7:**
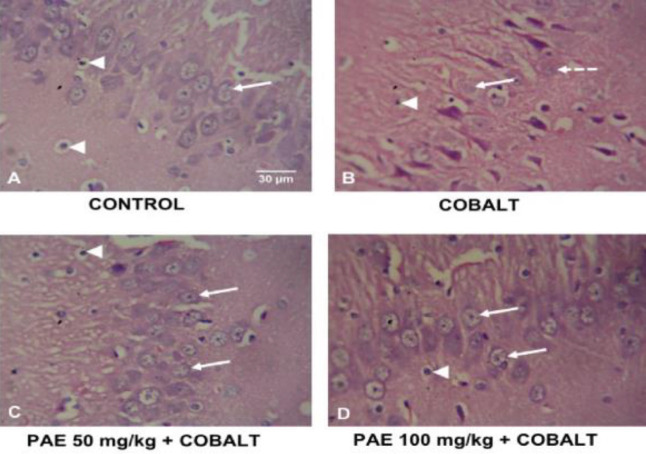
Photomicrographs of H&E-stained sections of the hippocampus (CA3 region) of control and treated rats. H&E x400. Scale bars: 50 mm. Arrows, intact neurons; Arrowheads, glial cells; Dashed arrows, degenerating neurons characterized by nuclear disintegration, nuclear shrinking, and cytoplasmic vacuolations

The Control group showed intact hippocampal histology. Large-sized pyramidal neurons are observed in the pyramidal layer of the CA3 fields. Large nuclei and conspicuous nucleoli characterize neurons. Many glial cells are visible as well. The cobalt-only group showed noticeable features of neurodegeneration characterized by nuclear disintegration, nuclear shrinking, and cytoplasmic vacuolations of the pyramidal cells. Treatment with PAE at 50 and 100 mg/kg showed improved hippocampal histology (Figure 7).

*Amygdala*

**Figure 8:**
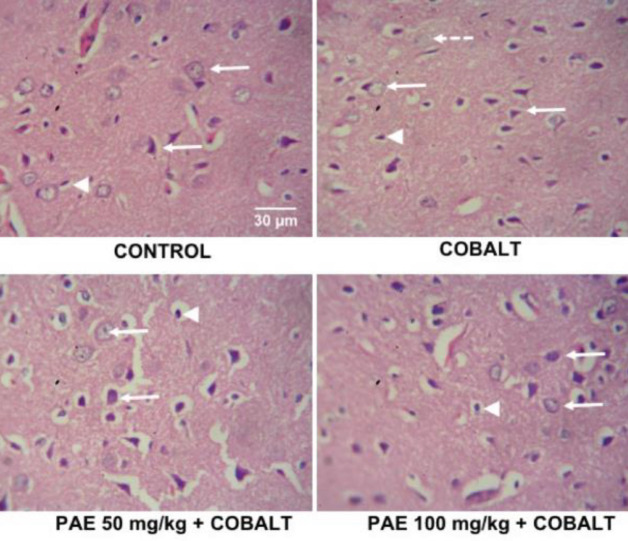
Photomicrographs of H&E-stained sections of the amygdala (basolateral) of control and treated rats. H&E x400. Scale bars: 50 mm. Arrows, intact neurons; Arrowheads, glia cells; Dashed arrows, degenerating neurons characterized by nuclear disintegration and cytoplasmic vacuolations

Control group shows intact histology of the amygdala with mostly medium-sized neurons, as well as glial cells. The Cobalt group showed mild neurodegenerating features with few neurons presenting as cytoplasmic vacuolations and nuclear disintegration. Treatment with PAE at 50 and 100 mg/kg showed mostly normal histology (Figure 8).

***Immunohistochemistry***

*Hippocampus*

**Figure 9:**
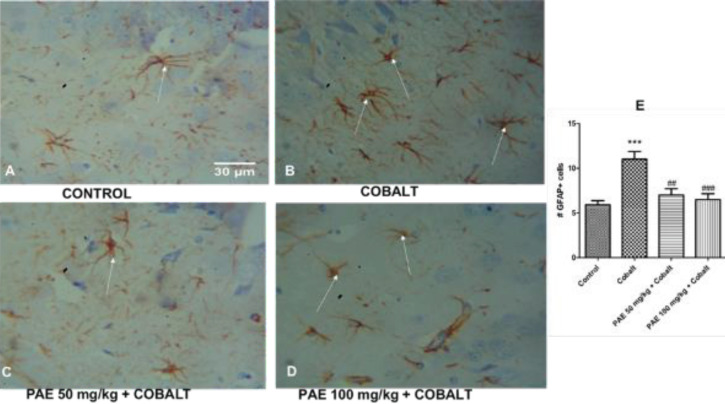
Photomicrographs of GFAP-stained sections of the hippocampus (CA3). Magnification: 400 x; Scale bars: 50 mm. The quantiﬁcation of GFAP-positive cells is shown in E. White arrows indicate GFAP-positive astrocytes ***P<0.001 compared with control, ##*P*<0.01, ### *P*<0.001 compared with cobalt only. One-way ANOVA followed by Tukey’s *post hoc* test

GFAP immunohistochemistry showed few astrocytes with fewer processes in the control group. On the other hand, the number of GFAP-positive astrocytes became more after receiving CoCl_2_. Rats co-treated with PAE showed similar effects, with cobalt only group having the most ramified effects, although this did not cut across all groups (A–D in Figure 9). Further analysis revealed that the number of GFAP-positive cells in CoCl_2_ and PAE-treated rats were significantly higher than in control rats (Figure 9E).

*Amygdala*

**Figure 10:**
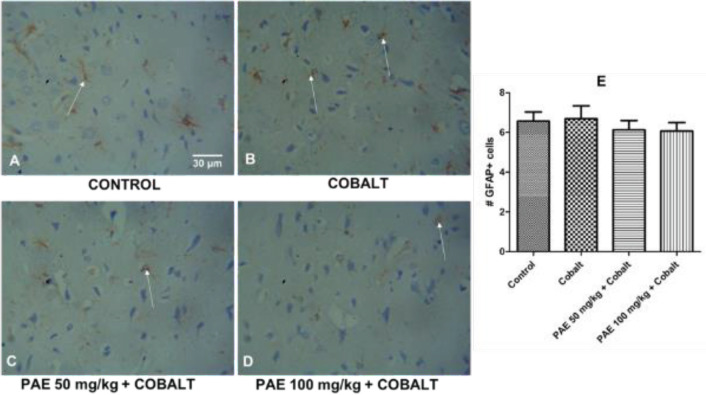
Photomicrographs of GFAP-stained sections of the amygdala (basolateral). Magnification: 400 x; Scale bars: 50 mm. The quantiﬁcation of GFAP-positive cells is shown in E. White arrows indicate GFAP-positive astrocytes ***P<0.001 compared with control, ##*P*<0.01, ###*P*<0.001 compared with cobalt only. One-way ANOVA followed by Tukey’s *post hoc* test

The control group presented with fewer GFAP-positive astrocytes. However, when exposed to cobalt, no obvious astrocytosis is observed, with little increase in astrocyte reaction. Similar effects are seen following PAE treatments. (A–D in Figure 10). Further analysis and quantification indicated that the number of GFAP-positive cells in CoCl_2 _and PAE-treated rats were not significantly different compared with the control group (Figure 10E).

PAE: Prosopis africana seed extract

PAE: Prosopis africana seed extract

PAE: Prosopis africana seed extract

PAE: Prosopis africana seed extract

PAE: Prosopis africana seed extract

PAE: Prosopis africana seed extract

GFAP: Glial fibrillary acidic protein

GFAP: Glial fibrillary acidic protein

## Discussion

Individuals who have been exposed to cobalt for a long time in the workplace and those who have received long-term cobalt chloride treatment for anemia have long been reported to have neurological difficulties (34). The revelation of elevated cobalt levels in the blood of people who have metal-on-metal hip prostheses has also reignited interest in cobalt toxicity. The current study looked at several toxicity targets in rats exposed to cobalt chloride and the mechanisms underpinning *PA* seed extract’s reversal of cobalt-induced neurotoxicity. Cobalt chloride altered the cytoarchitecture of the hippocampus and Amygdala, which *PA* seed extract attempted to alleviate in the current investigation. 

The present study used novel object recognition and Y-maze tests to assess short-term memory. In contrast, EPM was used to access amygdala-dependent or anxiety-related behavior in a CoCl_2_ rat model of hippocampal brain injury by evaluating exploration time, recognition index, % of correct alternation, open arm entries, and % time spent in open arms, respectively. The cobalt-only treated rats spent less time exploring the novel object, whereas the PAE-treated rats preferred the novel object and spent more time exploring it. The PAE-treated animals also had a better recognition index when compared with cobalt-only treated animals. The percentage of accurate alternation in the y-maze test was drastically reduced in cobalt-only treated rats suggesting that cobalt has a deleterious impact on cognitive capacity, resulting in memory loss. Previous research has linked memory and cognitive deficits to oxidative imbalance, with changes in anti-oxidant status causing oxidative stress and harming specific areas of the CNS, including the hippocampus, that are crucial for learning and memory (35, 36). On the other hand, co-administration with PAE resulted in a longer exploration time for the novel object and a higher recognition index. There was a more remarkable correct alternation comparable with the control group indicating that PAE may have the capacity to reverse the memory impairment produced by Cobalt exposure. Open-arm entry and time spent in open arms are classical parameters associated with anxiety, while time per open-arm entry provides an anxiolytic parameter not merely swayed by motor variables (37). The present study revealed that CoCl_2_-only treated animals spent significantly less time in the open arm and had lower open-arm entries. Suggesting that CoCl_2_ at the current dose could be anxiogenic as previous research revealed that an anxiogenic drug, caffeine, significantly decreased the time spent in the open arms of the elevated plus-maze (38). However, treatment with PAE resulted in increased open arm entries and duration comparable with that of the control group showing that PAE may have anxiolytic capabilities.  

As a result of its high oxygen consumption (the brain consumes around 20% of blood oxygen), low anti-oxidant enzyme activity, and the terminally orientated nature of its neurons, the brain is highly vulnerable to oxidative damage (39). More importantly, neuronal membranes are rich in polyunsaturated fatty acids and vulnerable to reactive oxygen species damage, resulting in changes in neuronal integrity and function (40). Induction of oxidative stress, on the other hand, is an often-used mechanism of cobalt poisoning (41). The degree of lipid peroxidation was determined by measuring MDA and H_2_O_2_ levels. The current study discovered a significant rise in H_2_O_2_ and MDA generation following cobalt administration. More so, cobalt administration also resulted in a decrease in the levels of essential anti-oxidant enzymes like SOD and GSH. Earlier research by (9) and (42) supports our findings since they both found elevated levels of H_2_O_2_ and MDA in the brains of rats after cobalt administration, as well as a contemporaneous reduction in GSH and SOD. However, treatment with PAE was shown to be beneficial in reversing cobalt-induced oxidative stress by increasing the activity of the anti-oxidant enzymes SOD and GSH while simultaneously lowering H_2_O_2_ and MDA levels. This finding is backed by (43) who had previously reported PAE’s anti-oxidant properties.

Neuronal injury is frequently exacerbated by metal-induced activation of specific glial cells, which results in the production of inflammatory mediators (44). Cobalt exposure resulted in a considerable increase in levels of the pro-inflammatory cytokines TNF-α and IL-1β in the current investigation. These findings are consistent with those of Mou *et al. *(44), who found a concentration- and time-dependent increase in TNF-α and IL-1β levels in N9 and primary mouse microglia after cobalt treatment, and Oria *et al.* who reported elevated levels of these proinflammatory biomarkers following CoCl_2_ administration (18). Additionally, our findings are consistent with those of (42), who found a significant increase in serum levels of TNF-α and IL-1β after cobalt administration. The pro-inflammatory effects of cobalt administration were dramatically reduced when treated with PAE.

Heavy metals like cadmium, nickel, and cobalt can influence the stimulation of numerous signal transduction pathways and form reactive radicals, which can lead to oxidative stress and mutagenesis, as well as lipid and protein degradation (45, 46). The present study revealed that the pyramidal neurons displayed apparent signs of neurodegeneration after cobalt administration, including nuclear disintegration, nuclear shrinkage, and cytoplasmic vacuolations. These features are in congruence with previous studies (47, 48) who reported substantial hippocampal alteration. Treatment with PAE, on the other hand, resulted in a considerable restoration of hippocampal histology. Similarly, exposure to cobalt chloride also affected the amygdala. The cobalt-only group showed neuronal deterioration, with neurons showing nuclear disintegration and cytoplasmic vacuolations. Treatment with PAE revealed mostly better histology, similar to the control group.

Glial fibrillary acidic protein (GFAP) is a protein in the intermediate filaments of glial cells commonly utilized as an astroglia cell marker. Glial cells play a significant role in neuroplasticity, and higher brain processes, hence abnormalities in astrocyte functionality are hallmarks in a growing number of illnesses (49). GFAP immunohistochemistry indicated reactive astroglia and increased expression of GFAP in the hippocampus after exposure to CoCl_2_ which agrees with a previous report (48). Increased GFAP expression indicates that this protein is up-regulated, which normally happens when brain tissues are injured (50). Nevertheless, following co-treatment with PAE, the activated astrocytes were reduced to values comparable with that of the control group. Additionally, there was reduced expression of GFAP in the PAE co-treated groups. In the same vein, astrocyte reactivity was apparent in the amygdala, especially in the cobalt-only group. However, the reactivity of astrocytes and expression of GFAP was not as pronounced as was observed in the hippocampus. Fewer GFAP-positive astrocytes, with small processes, were seen after treatment with PAE.

## Conclusion

Our findings show that cobalt-altered cognitive capabilities caused anxiogenic behaviors and altered hippocampo-amygdala neuronal histomorphology, likely via triggered oxidative stress, lowered anti-oxidant capacity, and neuroinflammation. On the other hand, *PA* seed extract negated cobalt-induced changes by suppressing damage to hippocampal and amygdala neurons, probably through its anti-oxidant and anti-inflammatory capabilities and consequently improving behavioral outcomes.

## Authors’ Contributions

RSO and OMI Designed the experiments; RBB, EPI, OLE, EGE, and OOE Performed experiments and collected data; RSO and UGE discussed the results and strategy; RSO and RBB Supervised, directed, and managed the study; OMI and RSO Approved the final version to be published.

## Conflicts of Interest

The authors declare no conflicts of interest.

## References

[1] Han DY, Hoelzle JB, Dennis BC, Hoffmann M (2011). A brief review of cognitive assessment in neurotoxicology. Neur Clin.

[2] Mason LH, Mathews MJ, Han DY (2013). Neuropsychiatric symptom assessments in toxic exposure. Psychiatr Clin North Am.

[3] Scharf B, Clement CC, Zolla V, Perino G, Yan B, Elci SG (2014). Molecular analysis of chromium and cobalt-related toxicity. Sci Rep.

[4] Cheyns K, Nkulu CBL, Ngombe LK, Asosa JN, Haufroid V, De Putter T (2014). Pathways of human exposure to cobalt in Katanga, a mining area of the DR Congo. Sci Total Environ.

[5] Mohamed AA-R, Metwally MM, Khalil SR, Salem GA, Ali HA (2019). Moringa oleifera extract attenuates the CoCl2 induced hypoxia of rat’s brain: expression pattern of HIF-1α, NF-kB, MAO and EPO. Biomed Pharmacother.

[6] Zhai Q, Narbad A, Chen W (2014). Dietary strategies for the treatment of cadmium and lead toxicity. Nutrients.

[7] Gonzales S, Polizio AH, Erario MA, Tomaro ML (2005). Glutamine is highly effective in preventing in vivo cobalt-induced oxidative stress in rat liver. World J Gastroenterol.

[8] Garoui EM, Fetoui H, Makni FA, Boudawara T, Zeghal N (2011). Cobalt chloride induces hepatotoxicity in adult rats and their suckling pups. Exp Toxicol Pathol.

[9] Garoui EM, Troudi A, Fetoui H, Soudani N, Boudawara T, Zeghal N (2012). Propolis attenuates cobalt induced-nephrotoxicity in adult rats and their progeny. Exp Toxicol Pathol.

[10] Clyne N, Hofman-Bang C, Haga Y, Hatori N, Marklund S, Pehrsson S (2001). Chronic cobalt exposure affects anti-oxidants and ATP production in rat myocardium. Scand J Clin Lab Invest.

[11] Achi O (2005). Traditional fermented protein condiments in Nigeria. Afr J Biotechnol.

[12] Ajiboye A, Agboola D, Fadimu O, Afolabi A (2013). Antibacterial, phytochemical and proximate analysis of Prosopis africana (Linn) seed and pod extract. FUTA J Res Sci.

[13] Olajide O, Fadimu O, Osaguona P, Saliman M (2013). Ethnobotanical and phytochemical studies of some selected species of leguminoseae of northern nigeria: a study of borgu local government area, niger state Nigeria. Int J Sci Nat.

[14] Erden-İnal M, Sunal E, Kanbak G (2002). Age-related changes in the glutathione redox system. Cell Biochem Funct.

[15] Uzodinma E, Mbaeyi-Nwaoha I, Onwurafor E (2020). Suitability of bacterial fermentation and foil packaging of condiment from African mesquite (Prosopis africana) seeds for nutritional retention and commercialization. Afr J Microbiol Res.

[16] Yusuf N, Ogah D, Hassan D, Musa M, Doma U (2008). Effect of decorticated fermented prosopis seed meal (Prosopis africana) on growth performance of broiler chicken. Int J Poult Sci.

[17] Council NR (2010). Guide for the care and use of laboratory animals.

[18] Oria RS, Obeten KE, Etetim AE, Mgbolu PE, Ikoku GI, Ijomone OM (2022). Ameliorative effect of prosopis africana seed extract on cobalt chloride induced cerebellar toxicity: Neurobehavioural, histomorphological and biochemical findings. Niger J Neurosci.

[19] Fedosova E, Shatskova A, Sarkisova KY (2021). Ethosuximide increases exploratory motivation and improves episodic memory in the novel object recognition test in WAG/Rij rats with genetic absence epilepsy. Neurosci Behav Physiol.

[20] Balogun WG, Morakinyo AO, Adeyemo KA, Imam A, Ishola A, Cobham A (2015). Neuroprotective potential of mango (magnifera indica) leave extract in alloxan-induced diabetic rats. Isra Med J.

[21] Abad S, Camarasa J, Pubill D, Camins A, Escubedo E (2016). Adaptive plasticity in the hippocampus of young mice intermittently exposed to MDMA could be the origin of memory deficits. Mol Neurobiol.

[22] Vaez Mahdavi MR, Roghani M, Khalili M, Dalir R (2010). The effect of food restriction on learning and memory of male Wistar rats: a behavioral analysis. Basic Clin Neurosci.

[23] Ijomone OM, Olaibi OK, Mba C, Biose IJ, Tete SA, Nwoha PU (2015). Chronic nicotine administration does not alter cognitive or mood associated behavioural parameters. Pathophysiology.

[24] Rao SS, Lago L, Volitakis I, Shukla JJ, McColl G, Finkelstein DI (2021). Deferiprone treatment in aged transgenic tau mice improves Y-maze performance and alters tau pathology. Neurotherapeutics.

[25] Sun W, Zhang L, Lu J, Yang G, Laundrie E, Salvi R (2008). Noise exposure–induced enhancement of auditory cortex response and changes in gene expression. Neuroscience.

[26] Knight P, Chellian R, Wilson R, Behnood-Rod A, Panunzio S, Bruijnzeel AW (2021). Sex differences in the elevated plus-maze test and large open field test in adult Wistar rats. Pharmacol Biochem Behav.

[27] Martin Jr JP, Dailey M, Sugarman E (1987). Negative and positive assays of superoxide dismutase based on hematoxylin autoxidation. Arch Biochem Biophys.

[28] Jollow D, Mitchell J, Zampaglione N, Gillette J (1974). Bromobenzene-induced liver necrosis Protective role of glutathione and evidence for 3, 4-bromobenzene oxide as the hepatotoxic metabolite. Pharmacology.

[29] Varshney R, Kale R (1990). Effects of calmodulin antagonists on radiation-induced lipid peroxidation in microsomes. Int J Rad Biol.

[30] Wolff SP (1994). [18] Ferrous ion oxidation in presence of ferric ion indicator xylenol orange for measurement of hydroperoxides. Meth Enzymol.

[31] Ijomone OM, Obi AU (2013). Kolaviron, isolated from garcinia kola, inhibits acetylcholinesterase activities in the hippocampus and striatum of wistar rats. Ann Neurosci.

[32] Akingbade GT, Ijomone OM, Imam A, Aschner M, Ajao MS (2021). D-ribose-l-cysteine improves glutathione levels, neuronal and mitochondrial ultrastructural damage, caspase-3 and GFAP expressions following manganese-induced neurotoxicity. Neurotox Res.

[33] Ijomone OM, Nwoha PU (2015). Nicotine inhibits hippocampal and striatal acetylcholinesterase activities, and demonstrates dual action on adult neuronal proliferation and maturation. Pathophysiology.

[34] Catalani S, Rizzetti M, Padovani A, Apostoli P (2012). Neurotoxicity of cobalt. Hum ExpToxicol.

[35] Omotoso GO, Mutholib NY, Abdulsalam FA, Bature AI (2020). Kolaviron protects against cognitive deficits and cortico-hippocampal perturbations associated with maternal deprivation in rats. Anat Cell Biol.

[36] Neves B-H, Menezes J, Souza MA, Mello-Carpes PB (2015). Physical exercise prevents short and long-term deficits on aversive and recognition memory and attenuates brain oxidative damage induced by maternal deprivation. Physiol Behab.

[37] Llano Lopez LH, Caif F, García S, Fraile M, Landa AI, Baiardi G (2012). Anxiolytic-like effect of losartan injected into amygdala of the acutely stressed rats. Pharmacol Rep.

[38] Ansah C, Mfoafo EA, Woode E, Duwiejua M (2008). Anxiogenic effects of an aqueous crude extract of Cryptolepis sanguinolenta (periplocaeaeceae) in mice. Int J Pharmacol.

[39] Dringen R, Gutterer JM, Hirrlinger J (2000). Glutathione metabolism in brain: metabolic interaction between astrocytes and neurons in the defense against reactive oxygen species. Eur J Biochem.

[40] Mates J (2000). Effects of anti-oxidant enzymes in the molecular control of reactive oxygen species toxicology. Toxicology.

[41] Catelas I, Petit A, Vali H, Fragiskatos C, Meilleur R, Zukor DJ (2005). Quantitative analysis of macrophage apoptosis vs necrosis induced by cobalt and chromium ions in vitro. Biomaterials.

[42] Akinrinde A, Adebiyi O (2019). Neuroprotection by iuteolin and gallic acid against cobalt chloride-induced behavioural, morphological and neurochemical alterations in wistar rats. Neurotoxicology.

[43] Ugwu M, Asuk A, Utu-Baku A, Eteng M (2018). Tissue-protective effect of Prosopis africana seed extract on testosterone and estradiol induced benign prostatic hyperplasia of adult male rats. Int J Innov Res Adv Stud.

[44] Mou YH, Yang JY, Cui N, Wang JM, Hou Y, Song S (2012). Effects of cobalt chloride on nitric oxide and cytokines/chemokines production in microglia. Int Immunopharmacol.

[45] Valko M, Morris H, Cronin M (2005). Metals, toxicity and oxidative stress. Curr Med Chem.

[46] Ognjanović BI, Marković SD, Pavlović SZ, Žikić RV, Štajn A (2008). Effect of chronic cadmium exposure on anti-oxidant defense system in some tissues of rats: Protective effect of selenium. Physiol Res.

[47] Adebiyi OE, Olopade JO, Olayemi FO (2018). Sodium metavanadate induced cognitive decline, behavioral impairments, oxidative stress and down regulation of myelin basic protein in mice hippocampus: Ameliorative roles of β-spinasterol, and stigmasterol. Brain Behav.

[48] Oria R, Okafor A, Anyanwu G (2021). Histomorphological changes in hippocampus of adult albino Wistar rat following administration of graded doses of cobalt. J Sci Eng Technol.

[49] Allaman I, Bélanger M, Magistretti PJ (2011). Astrocyte–neuron metabolic relationships: For better and for worse. Trends Neurosci.

[50] Ekong MB, Ekpene UU, Nwakanma AA, Bello EF (2017). The combination of the extracts of Rauwolfia vomitoria and Gongronema latifolium show protective effects on the cerebellum. Synergy.

